# Extracting Quality of Life Information of Patients Diagnosed With Breast Cancer From Health Care Online Forum Posts: Data Feasibility Study

**DOI:** 10.2196/76044

**Published:** 2026-04-30

**Authors:** David Maria Schmidt, Raoul Schubert, Brian Po-Han Chen, Deborah Kuk, Valmeek Kudesia, Andreas Hinz, Philipp Cimiano

**Affiliations:** 1 Center for Cognitive Interaction Technology, Faculty of Technology, Bielefeld University, Inspiration 1, Bielefeld, 33619, Germany, 49 521 106-12008; 2 Inspire, Arlington, VA, United States; 3 Department of Medical Psychology and Medical Sociology, University of Leipzig, Leipzig, Germany; 4 Semalytix GmbH, Bielefeld, Germany

**Keywords:** quality of life, social media listening, patient-focused drug development, health care communities, breast cancer

## Abstract

**Background:**

Quality of life (QoL) questionnaires are used in many disease areas to measure the burden that a disease causes for patients, which help provide insights into disease impact, identify unmet medical needs, and inform patient-centered drug development and value assessment for treatments. The collection of data imposes both a significant burden on patients as well as effort on health care personnel, thus incurring high costs for the health care system. Given that patients share detailed information about their condition and treatment experiences on social media and patient forums, an important research question is to what extent information about QoL can be obtained from patients’ online forum posts to potentially complement information obtained from questionnaires.

**Objective:**

This study aimed to assess how much QoL information can be gained from the analysis of posts by patients in online health care communities and whether this information is rich enough to estimate individual patient’s QoL based on their posts. We conducted this feasibility study in the context of breast cancer as it is the most prevalent cancer in the female population.

**Methods:**

We recruited 134 female patients diagnosed with breast cancer on the Inspire patient online forum, who voluntarily participated in our feasibility study. They filled in the EORTC (European Organisation for Research and Treatment of Cancer) QLQ-C30 and QLQ-BR23 questionnaires consisting of 30 general questions and 23 additional breast cancer–specific questions and provided consent to analyze their posts and comments on the online forum (756 posts and 19,478 comments). Posts were coded manually to identify parts of the text providing answers to 1 of the aforementioned 53 questions.

**Results:**

The data annotation yielded a substantial agreement (mean Fleiss κ of 0.5, SD 0.28). Overall, we found answers in the coded data for 50 out of 53 EORTC QLQ-C30 and QLQ-BR23 questions. The information coded in the posts reliably predicted the answers given in the questionnaires (*F*_1_-score=0.7), with even better results when grouping similar questions (*F*_1_-score=0.8 for fine-grained and 0.9 for coarse-grained grouping). The 5 questions that were most frequently answered on the basis of the coded posts were “Did you feel ill or unwell?” (304 of 2683 annotated posts and comments), “Did you worry?” (105 posts and comments), “Have you had pain?” (104 posts and comments), “Did you feel tense?” (85 posts and comments), and “Were you limited in doing either your work or other daily activities?” (77 posts and comments).

**Conclusions:**

Our feasibility study shows that there is valuable QoL-related information in posts of online patient communities, which can potentially serve as an innovative low-burden QoL monitoring approach. Future research should consider how these insights can be used to complement existing QoL instruments and whether the process of extracting QoL-related information can be automated.

## Introduction

Quality of life (QoL), as defined by the World Health Organization, encompasses physical, mental, and social well-being beyond the mere absence of disease [1]. Assessing QoL is essential in health care, providing insights into disease impact, identifying unmet medical needs, and informing patient-centered drug development and value assessment for treatments. Traditionally, QoL is measured using patient-reported outcome questionnaires, such as the widely used EORTC (European Organisation for Research and Treatment of Cancer) QLQ-C30 for patients with cancer [2]. These standardized tools facilitate comparisons across patient groups and with the general population.

However, QoL assessments are often time-consuming and administratively burdensome for both patients and health care providers [3-7]. Efforts to streamline data collection have explored digital and computer-supported approaches [6-8]. Given the increasing use of social media and online patient communities for sharing health experiences, a key question arises: Can patient-generated online content serve as a proxy or complementary source for QoL data collection? While not a replacement for structured surveys, social media data may provide real-time, cost-effective insights into patient well-being, preferences, and treatment experiences.

Previous research has leveraged social media to assess disease burden and treatment experiences across conditions, such as Parkinson disease [9,10], Sjögren disease [11], chronic obstructive pulmonary disease [12], and mental disorders [13,14]. Some related work also analyzed patient posts compared to a specific QoL ground truth, but on an aggregated level. For example, Zivanovic et al [15] compared Twitter data with official QoL data of the city of Bristol. Similarly, Cotté et al [16] also investigated cancer-specific forums and QoL data, but with a focus on immune checkpoint inhibitors and not the QoL impact for individual users. Therefore, studies have yet to systematically evaluate whether individual QoL data can be reliably extracted from online patient discussions.

To explore this, we conducted a study with patients diagnosed with breast cancer from the Inspire patient online forum, assessing whether online forum posts contain relevant QoL information. Breast cancer remains the most commonly diagnosed cancer in women, with over 2.3 million new cases in 2020 [17]. QoL is a critical concern for patients diagnosed with breast cancer and survivors, who frequently experience significant impairments [18-21].

In our study, patients diagnosed with breast cancer completed standard QoL questionnaires (EORTC QLQ-C30 and QLQ-BR23), while their online posts and comments were manually coded to determine the presence of QoL-related content. We then assessed the prevalence of QoL discussions and their correlation with questionnaire responses. Our research focuses on the following questions:

- RQ1: Do patient online forum posts contain QoL-related information?
- RQ2: Which QoL questions from EORTC QLQ-C30 and QLQ-BR23 are most frequently addressed in online posts?
- RQ3: How well do these online discussions align with individual patients’ QoL questionnaire responses?

This study aims to determine whether patient-generated content from online health communities can complement traditional QoL assessments, potentially offering a scalable, low-burden alternative for understanding patient well-being.

## Methods

### Overall Structure

**Figure 1.**
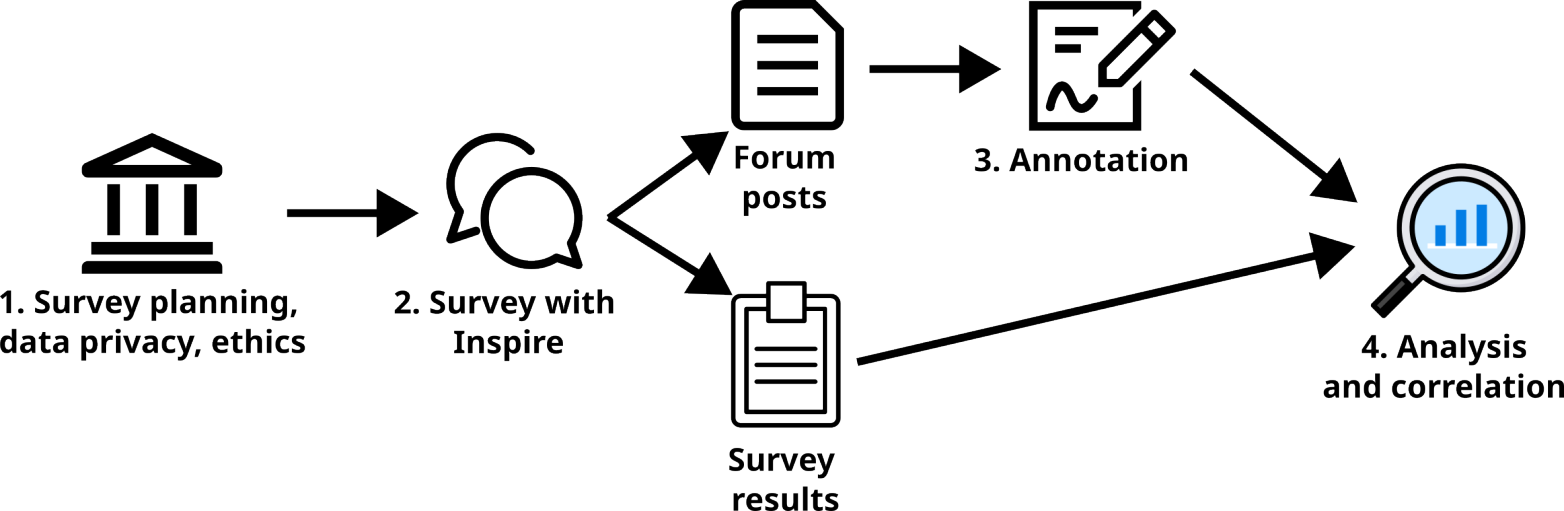
Overview of the study workflow from planning survey conduction to data analysis. The online survey using the EORTC QLQ-C30 and QLQ-BR23 questionnaires was conducted between May and June 2024 with patients diagnosed with breast cancer posting in the Inspire patient online forums. EORTC: European Organisation for Research and Treatment of Cancer.

This feasibility study aimed to assess the presence of QoL information in online discussions by patients diagnosed with breast cancer. Breast cancer was selected due to its high prevalence, and data were collected from patient-generated content on the Inspire patient online forum. Patient communities on this forum are organized by condition, including “breast cancer” and “advanced breast cancer,” where each thread of natural peer-to-peer conversation consists of an initial post and subsequent comments. An overview of the workflow followed in this study is given in Figure 1.

To compare patient-reported QoL in online posts with structured assessments, we (the authors of this study) administered the QLQ-C30 questionnaire by the EORTC [2] and its breast cancer–specific module, QLQ-BR23. These validated instruments provided a reference (ground truth) against which the content of online discussions could be analyzed.

Participants were recruited from the “Breast Cancer” and “Advanced Breast Cancer” communities on Inspire. Eligibility criteria included female patients diagnosed with breast cancer, age 18 years or older, residency in the United States, and at least 1 post or comment in the respective Inspire communities.

Residency in the United States was included due to Inspire being based in the United States.

The EORTC QLQ-C30 questionnaire was used to assess general QoL in patients with cancer. It comprises 30 items grouped into 5 functioning scales, a global health status/QoL scale, 3 symptom scales, and 6 single-item measures. Scores range from 0 to 100, with higher functioning scores and lower symptom scores indicating better QoL. Normative values for this instrument have been previously established [22,23]. The breast cancer–specific module, EORTC QLQ-BR23, consists of 23 items categorized into 8 scales: systemic therapy side effects, hair loss, arm symptoms, breast symptoms, body image, future perspective, sexual functioning, and sexual enjoyment. Response options align with those of the EORTC QLQ-C30.

The final survey also included demographic questions, such as year of birth, US state of residence, gender, sex at birth, insurance type, and residential setting (urban, suburban, rural, or other). The survey required approximately 5 to 10 minutes to complete and was conducted between May and June 2024.

### Ethical Considerations

Our study has been approved by the Ethics Committee of Bielefeld University under application number 2023‐216-W1. A filled CHERRIES (Checklist for Reporting Results of Internet E-Surveys) [24] checklist can be found in Checklist 1. Informed consent was obtained from all participants, granting permission to analyze their posts and comments for this study. The collected data, especially the survey responses, are not publicly shared and only available to the authors of the study. Additionally, the data are stored in encrypted form only. All eligible community members were invited to participate (ie, convenience sampling), with the first 100 respondents receiving a US $20 gift card as compensation.

### Annotation and Coding Process

The objective of the coding process was to identify text spans within collected posts and comments that could correspond to responses in the EORTC QoL questionnaires. To achieve this, we implemented an annotation schema similar to named-entity recognition, where each of the 53 QoL questions served as a label. Unlike traditional named-entity recognition, which identifies entities, such as persons or organizations [25,26], our approach used QoL-related labels to tag relevant text segments.

In addition to assigning labels corresponding to each QoL question, we introduced supplementary labels to capture both reported presence and absence of symptoms: “positive” or “negative.” Coders applied these labels alongside question-specific tags to indicate whether a response reflected a favorable or unfavorable QoL experience. Furthermore, an “Exact match” label was used to denote text spans that provided precise or highly relevant answers, while standard labels without this addition were applied to responses that were partial or vague.

The annotation schema and guidelines were developed in a 2-phase process. In the first phase, 3 annotators labeled 30 posts using a preliminary schema. After completing this pilot phase, the results were reviewed, and minor refinements were made. For instance, the label “Did you feel ill or unwell?” was designated for general QoL descriptions that did not fit into other, more specific categories. The annotators were explicitly trained to only use the fallback label when they could not find any other label that fits better, and this was also added to the annotation guidelines (Multimedia Appendix 1). Compliance with these rules was ensured by closely checking fallback annotations during the beginning of the annotation process of the full data. These pilot annotations were subsequently excluded from the final dataset.

In the second phase, the full annotation process commenced, with no further modifications needed. Additionally, posts and comments from the last 6 months (relative to each user’s participation date) were labeled to indicate whether they referred to past events. This distinction was critical for evaluating the correlation between forum data and survey responses, as older event descriptions may not accurately reflect a patient’s current QoL. Filtering out such posts allowed for a more precise alignment between forum-derived insights and survey-based QoL assessments.

### Measuring Information Consistency Between Online Posts and Survey Responses

To assess the alignment between information shared in online posts and responses in the structured questionnaires, we applied classification metrics commonly used in machine learning. The coding scheme and questionnaire responses were simplified into a binary format, mapping each piece of information from posts and survey answers to either a “positive” or “negative” response. The intended meaning of “positive” here is that the corresponding question would be answered with “yes,” thus indicating the presence of symptoms or other issues. Analogously, “negative” corresponds to answering the question with “no,” indicating the absence of symptoms or other issues. Specifically, the answer categories “a little,” “quite a bit,” and “very much” were classified as “positive,” whereas “not at all” was categorized as “negative.” This enabled the computation of key classification metrics as follows:

- True positives (TP): Instances where both the online post and the corresponding questionnaire response indicate a “positive” answer (eg, a user stating, “I had so much pain last week!” and selecting “yes” for the survey question, “Have you had pain?”).
- False positives (FP): Cases where an online post is labeled as “positive,” but the corresponding survey response is “negative” (eg, a user stating, “I am so worried!” but responding “no” to the question, “Did you worry?”).
- True negatives (TN): Instances where both the online post and the survey response indicate a “negative” answer (eg, a user stating, “I do not have any pain!” and selecting “no” for the survey question, “Have you had pain?”).
- False negatives (FN): Cases where an online post is labeled as “negative,” but the corresponding survey response is “positive” (eg, a user stating, “I did not worry at all last week!” but selecting a “positive” response in the survey question, “Did you worry?”).

If no labeled data existed for a specific user and QoL question, that user did not contribute to the TP, FP, TN, or FN calculations for that question. This approach allowed us to evaluate how well the available data could predict survey responses.

Using the TP, FP, TN, and FN values, we computed standard machine-learning classification metrics, including precision (TP/[TP + FP]), recall (TP/[TP + FN]), and *F*_1_-score as harmonic mean between precision and recall, that is, 2× precision × recall/(precision + recall) [27]. Given that data sparsity increases when analyzing results at the individual user level across all 53 survey questions, we explored grouping similar survey questions based on the EORTC scoring manual. This resulted in 2 levels of categorization: (1) fine-grained grouping: based on detailed QoL question clusters (Table 1) and (2) coarse-grained grouping: functional (questions 1-7, 20-27, 39-46), symptoms (questions 8-19, 28, 31-38, 47-53), and global health (questions 29 and 30).

These groupings enabled more robust analyses by reducing sparsity and improving interpretability of the correspondence between online forum discussions and structured survey responses.

**Table 1.** Fine-grained grouping based on the EORTC^a^ QLQ-C30 and QLQ-BR23 scoring manual^b^.

Group	Question IDs
Global health	29, 30
Physical functioning	1, 2, 3, 4, 5
Role functioning	6, 7
Emotional functioning	21, 22, 23, 24
Cognitive functioning	20, 25
Social functioning	26, 27
Fatigue	10, 12, 18
Nausea and vomiting	14, 15
Pain	9, 19
Dyspnea	8
Insomnia	11
Appetite loss	13
Constipation	16
Diarrhea	17
Financial difficulties	28
Systemic therapy side effects	31, 32, 33, 34, 36, 37, 38
Upset by hair loss	35
Arm symptoms	47, 48, 49
Breast symptoms	50, 51, 52, 53
Body image	39, 40, 41, 42
Future perspective	43
Sexual functioning	44, 45
Sexual enjoyment	46

a EORTC: European Organisation for Research and Treatment of Cancer.

b Question IDs refer to the corresponding EORTC questionnaire question numbers. The online survey using the EORTC QLQ-C30 and QLQ-BR23 questionnaires was conducted between May and June 2024 with patients diagnosed with breast cancer posting in the Inspire patient online forums.

## Results

### General Statistics

**Figure 2.**
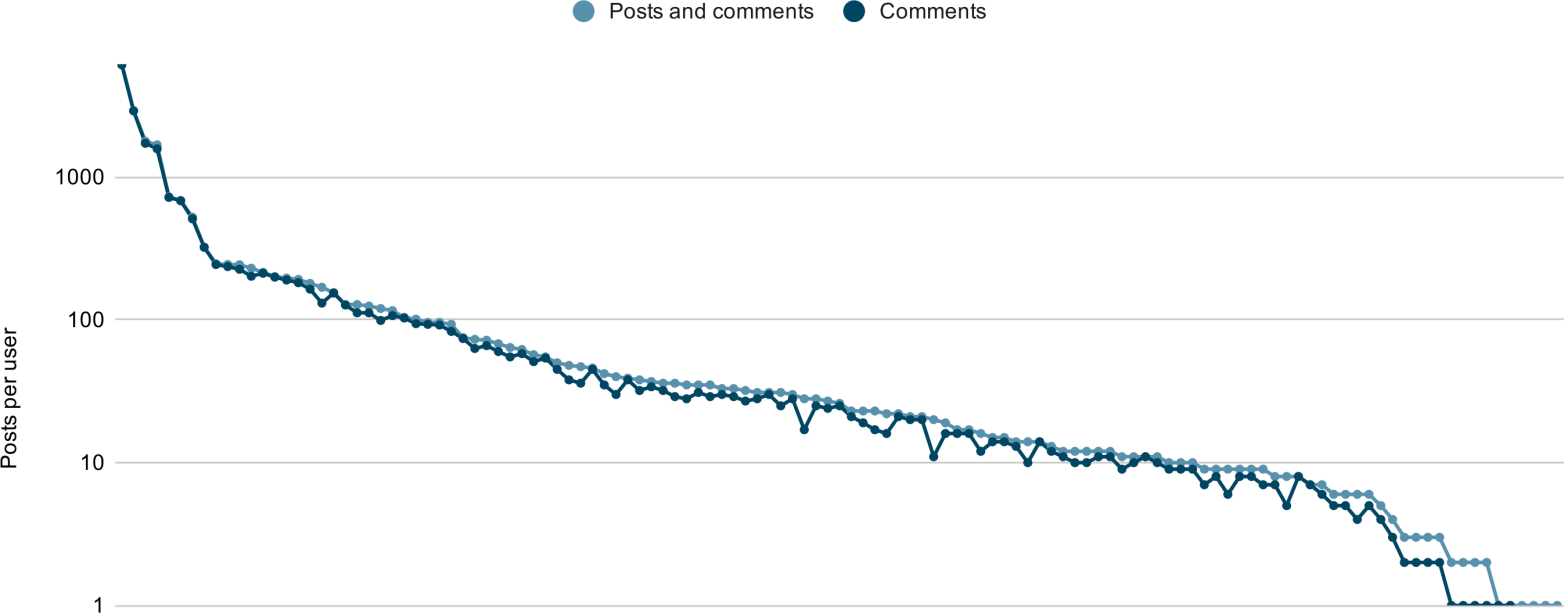
Posts per user, with 123 users on the x-axis and the (log-scaled) number of posts on the y-axis, differentiating between combined posts and comments in light blue and comments only in dark blue. Users without posts or comments were disregarded. The online survey using the EORTC QLQ-C30 and QLQ-BR23 questionnaires was conducted between May and June 2024 with patients diagnosed with breast cancer posting in the Inspire patient online forums. EORTC: European Organisation for Research and Treatment of Cancer.

The collected dataset comprises 20,204 posts and comments from 2006 to 2024, with 580 posts and comments originating within the 6 months preceding the end of the study. The distribution of posts per user follows a long-tail pattern, as illustrated in Figure 2, with absolute values presented in Table 2). As shown in Figure 2, the majority of contributions are comments rather than original posts. Table 3 shows that our data cover a broad spectrum of patients diagnosed with breast cancer, both in terms of age and distance between survey and breast cancer diagnosis.

**Table 2.** Overview of the number of posts per user^a^.

Number of posts and comments	Number of users
1	6
2-10	28
11-100	63
101-1000	22
>1000	4

a The online survey using the EORTC QLQ-C30 and QLQ-BR23 questionnaires was conducted between May and June 2024 with breast cancer patients posting in the Inspire patient online forums.

The average post length is 408.4 (SD 366.8) characters. A total of 134 participants were enrolled in the study, of whom 11 did not contribute at least 1 post or comment, resulting in 123 participants meeting the inclusion criteria.

For annotation, the last 6 months of data (580 posts and comments) were labeled by 3 annotators, with an average annotation time of 15.9 (SD 10.3) hours per annotator. The last 24 months of data (an additional 2103 posts and comments) were labeled by 2 annotators, requiring an average of 76.5 (SD 12.7) hours per annotator. In total, 2683 posts were annotated. Fourteen posts contained QoL-related information referring to someone other than the posting user. These posts were annotated but excluded from statistical evaluation.

Of the 2669 annotated posts and comments included in the final dataset, 613 (23%) contained at least 1 label corresponding to a QoL question. When considering only the last 6 months, the proportion was higher at 37.4% (217 out of 580 posts), indicating that approximately one-third to one-fourth of all posts contained QoL-relevant content.

**Table 3.** Statistics of self-reported demographic data of survey participants^a^.

Data category	Mean (SD)	Minimum	Maximum
Age	68.2 (11.5)	27	116
Age at diagnosis	55.7 (12.6)	26	103
Years since diagnosis	13.1 (9.4)	0	45

a The online survey using the EORTC QLQ-C30 and QLQ-BR23 questionnaires was conducted between May and June 2024 with patients diagnosed with breast cancer patients posting in the Inspire patient online forums.

Among the 53 QoL questions, 50 were labeled at least once, with the most frequently occurring questions summarized in Table 4. The most commonly assigned label was “Did you feel ill or unwell?,” with 301 annotations, representing 11% (n=301) of all posts and 57.3% (n=47) of users who actively posted during the study period. It has to be noted that this is used as a fallback label for the case where posts or comments contain information which is clearly QoL-relevant, but no other better-fitting label could be found. An example for this is when patients describe some very specific or very vague symptoms that do not correspond well to any other question (eg, “the symptoms increased a lot”). The next most frequently annotated questions were “Did you worry?” (105 annotations) and “Have you had pain?” (102 annotations), both capturing broad categories of patient discomfort. The overall label distribution is visualized in Multimedia Appendix 2.

**Table 4.** Top 10 most frequent labels with respect to number of posts they are annotated in for all annotated posts (most recent 24 months)^a^.

Question	Number of posts	Portion of posts, %	Number of users	Portion of users, %
Did you feel ill or unwell?	301	11	47	57.3
Did you worry?	105	3.9	26	31.7
Have you had pain?	102	3.7	32	39
Did you feel tense?	84	3.1	21	25.6
Were you limited in doing either your work or other daily activities?	77	2.8	18	22
Were you tired?	55	2	18	22
Have you felt nauseated?	51	1.9	18	22
Were you worried about your health in the future?	41	1.5	16	19.5
Have you lacked appetite?	32	1.2	5	6.1
Did you feel depressed?	32	1.2	14	17.1

a “Portion of posts” refers to the percentage of posts with the corresponding label relative to all posts of that time period. Similarly, “Portion of users” refers to the number of users who have posts annotated with that label relative to all users who posted in that time period. The online survey using the European Organisation for Research and Treatment of Cancer (EORTC) quality of life questionnaires (QLQ) QLQ-C30 and QLQ-BR23 was conducted between May and June 2024 with patients diagnosed with breast cancer posting in the Inspire patient online forums.

#### Annotator Agreement

As every post and comment within the most recent 2 years was annotated by 2 or 3 annotators, the interannotator agreement can be calculated via Fleiss κ values [28]. This measure aids in determining to which degree annotators agreed on which text segments or posts answered that question at least partially. We only consider the basic labels corresponding to the questions for the calculation of interannotator agreement, ignoring the additional labels “negative” and “exact match,” and handle each question separately, as this fits best with the setting assumed by the Fleiss κ metric for interannotator agreement.

We calculated the Fleiss κ for 2 different levels of annotation granularity, that is, on word and on post or comment level, respectively. The word level reflects the actual annotations, that is, the text segments created during annotation, whereas the post level is a simplification aggregating all annotations of a post such that the annotations only have to agree on whether the whole text answers a specific question or not, but not where exactly.

The top and bottom 10 Fleiss κ values for the most recent 6 months of data are provided in Table 5, together with the absolute numbers of posts in which the corresponding question has been answered.

It can be observed that many frequently occurring labels exhibit solid to high annotator agreement, for example, “Have you lost any hair?” has a Fleiss κ value of 0.7 for word units and even 1.0 for post units while being present in 26 posts in total. Even the fallback label “Did you feel ill or unwell?” with 285 labeled posts has Fleiss κ values of 0.6 and 0.7 for word and post granularity, respectively.

Most questions in the lower range of Fleiss κ values have a very low number of labeled posts. The 6 questions with the lowest observed agreement of 0.0 together only account for 14 posts in total, so these appear to be cases where the annotators do not agree whether the label is applicable. Further investigating the data, the most disagreement is caused by vaguely formulated posts for which a label could but does not necessarily have to apply. For example, for labels, such as “Did you have any pain in your arm or shoulder?,” issues can range from just vaguely described body areas to mentions of certain diseases or syndromes, which are closely connected to pain in that area, without pain being explicitly mentioned. Although we cannot give real posts as examples for data privacy reasons, we summarized prototypical posts to illustrate the reasons for disagreement. For “Did you have any pain in your arm or shoulder?,” annotators disagreed for posts, such as “I take that medication for 5 years now and started to have problems with hand-foot syndrome some time ago,” as hand-foot syndrome itself is not an explicit mention of pain and can occur with or without pain [29]. Similarly, annotators disagreed for posts, such as “In the last months I had cramps in different parts of my hand from time to time.” Another label with low annotator agreement was “Have you had skin problems on or in the area of your affected breast (e.g., itchy, dry, flaky)?,” for example, for posts like “I try to avoid foods with high folic acid as much as possible as they can cause pain in the arms for some of us. For the dryness, I use some ointment all the time,” as it is not very clear what part of the body “dryness” refers to.

**Table 5.** Annotator agreement for the 10 questions with the highest agreement and 10 questions with the lowest agreement (Fleiss κ, the most recent 6 months of data before the survey) using word units and post units^a^.

Question	Fleiss κ (word units)	Fleiss κ (post units)	Labeled posts
Top 10			
Did you have headaches?	1.0	1.0	3
Have you had difficulty remembering things?	0.9	1.0	3
Have you lacked appetite?	0.9	0.9	15
Did you have hot flushes?	0.8	0.8	9
Have you lost any hair?	0.7	1.0	26
To what extent were you sexually active? (with or without intercourse)	0.7	1.0	6
Did you feel ill or unwell?	0.6	0.7	285
Have you had diarrhea?	0.6	0.9	10
Do you have any trouble taking a short walk outside of the house?	0.6	0.7	19
Were you tired?	0.6	0.8	35
Bottom 10			
Have you had skin problems on or in the area of your affected breast (eg, itchy, dry, and flaky)?	0.2	0.3	11
Have you vomited?	0.2	0.5	2
Do you have any trouble taking a long walk?	0.2	0.3	7
Did you have any pain in your arm or shoulder?	0.2	0.4	11
How would you rate your overall health during the past week?	0.0	0.0	1
Did you need to rest?	0.0	0.0	1
Was it difficult to raise your arm or to move it sideways?	0.0	0.0	1
Did pain interfere with your daily activities?	0.0	0.0	3
Did food and drink taste different than usual?	0.0	0.0	6
How would you rate your overall quality of life during the past week?	0.0	0.0	2

a The online survey using the European Organisation for Research and Treatment of Cancer (EORTC) quality of life questionnaires (QLQ) QLQ-C30 and QLQ-BR23 was conducted between May and June 2024 with patients diagnosed with breast cancer posting in the Inspire patient online forums.

### Correlation Results Between Annotated Data and Survey Data

We estimate the correlation between the information provided by the authors to QoL aspects and the data provided in the questionnaires by computing *F*_1_-score as described in the “*Measuring Information Consistency Between Online Posts and Survey Responses*” section. Table 6 shows the *F*_1_-scores and total annotations for the 10 most frequently annotated questions based on data originating from the most recent 6-month period and compares these numbers to the corresponding values for 24 months.

As observed from Table 6, all of the 10 most frequently answered questions can be predicted reliably with an *F*_1_-score of 0.6 or higher for the last 6 months of data. For 7 of the 10 questions, the *F*_1_-score is even 0.8 or higher. The lowest score of 0.6 is achieved for the most frequent question, that is, “Did you feel ill or unwell?” This specific question is also used as a fallback label for utterances, which do not fit in any other category, thus increasing prediction difficulty compared to other labels. Even when considering a much larger time period of 24 months, the *F*_1_-scores for these questions do not drop below 0.5, with some even increasing.

Although the observed *F*_1_-scores are high for many questions, for example, “Were you tired?” or “Were you worried about your health in the future?” with each of an *F*_1_-score of 1.0, for some questions, the available data get sparse and the data sparsity increases even further when considering single users or shorter time spans, leading to lower *F*_1_-scores in some cases, for example, for “Have you vomited?” (*F*_1_-score of 0.2 with 2 labeled posts in 6 months of data). One way of reducing data sparsity is to consider a larger time span. However, this comes at the cost of posts and comments with a larger temporal distance from the survey date being potentially less relevant with respect to the QoL at that time. However, in Table 6, despite the inclusion of older posts, we can observe solid to high *F*_1_-scores (0.6-1.0, mean 0.8, SD 0.1) for all 10 most frequently answered questions. This shows that including posts from dates outside the time period asked for in the QoL surveys can still be useful.

**Table 6.** *F*_1_-scores for the 10 most frequently annotated questions, describing how well annotations predict the actual answers given in the survey, considering the last 6 and 24 months of data, which are not filtered by time deviation^a^.

Question	*F*_1_-score (6 mo)	Total annotations (6 mo)	*F*_1_-score (24 mo)	Total annotations (24 mo)
Did you feel ill or unwell?	0.6	127	0.7	222
Did you worry?	0.8	36	0.9	47
Did you feel tense?	0.9	33	0.8	40
Have you had pain?	0.8	28	0.8	74
Were you tired?	1.0	14	0.9	50
Have you felt nauseated?	0.7	14	0.5	34
Has your physical condition or medical treatment interfered with your social activities?	0.7	13	0.7	13
Has your physical condition or medical treatment caused you financial difficulties?	1.0	12	1.0	12
Were you worried about your health in the future?	1.0	11	1.0	15
Have you lost any hair?	0.8	10	0.8	29

a The online survey using the European Organisation for Research and Treatment of Cancer (EORTC) quality of life questionnaires (QLQ) QLQ-C30 and QLQ-BR23 was conducted between May and June 2024 with patients diagnosed with breast cancer posting in the Inspire patient online forums.

As some questions occur sparsely, we group the answers from the posts and the questionnaires into the groups “Symptomatic” and “Functional” used in the EORTC scoring manual. The results for the grouped questions are shown in Table 7.

The only group in Table 7 having annotations available and exhibiting a lower *F*_1_-score than 0.7 for the last 6 months is “cognitive functioning,” which comprises only 2 questions, that is “Have you had difficulty in concentrating on things, like reading a newspaper or watching television?” and “Have you had difficulty remembering things?” All other groups show very promising results, with over half of the groups having scores of 0.9 or higher. Groups of questions for which there were no annotations in the data, that is “Appetite loss,” “Global health,” “Insomnia,” “Sexual enjoyment,” “Sexual functioning,” and “Upset by hair loss,” have been omitted in the table. Even when considering much older data from the last 24 months as well, *F*_1_-scores do not drop below 0.5, with many scores even increasing, underlining the relevance of older data.

For 6 months of data, a total of 31 users have shared posts containing QoL information with on average 10.8% (SD 9.8%) of the 53 QoL questions answered. For the full 24 months of annotated data, there are 46 users with QoL-relevant posts, with 10.8% (SD 10.1%) of the questionnaire questions answered on average.

**Table 7.** *F*_1_-scores describing how well annotations predict the actual answers given in the survey, considering the last 6 and 24 months of data, using fine-grained and coarse-grained groups, which are filtered by time deviation^a^.

Question	*F*_1_-score (6 mo)	Total annotations (6 mo)	*F*_1_-score (24 mo)	Total annotations (24 mo)
Fine grouping				
Fatigue	1.0	5	0.9	44
Future perspective	1.0	5	1.0	9
Arm symptoms	1.0	4	1.0	6
Diarrhea	1.0	3	0.6	16
Dyspnea	1.0	2	0.9	14
Nausea and vomiting	1.0	2	0.6	25
Body image	1.0	1	1.0	1
Financial difficulties	0.9	9	0.9	9
Emotional functioning	0.9	38	0.9	70
Social functioning	0.9	11	0.9	11
Physical functioning	0.9	9	0.8	12
Breast symptoms	0.9	7	0.9	11
Role functioning	0.8	10	0.7	21
Systemic therapy side effects	0.7	59	0.9	189
Pain	0.7	15	0.8	61
Constipation	0.7	2	0.8	10
Cognitive functioning	0.0	1	0.5	3
Insomnia	—^b^	0	1.0	1
Appetite loss	—	0	0.7	16
Coarse grouping				
Functional	0.9	68	0.9	120
Symptomatic	0.8	101	0.9	385

a The online survey using the EORTC QLQ-C30 and QLQ-BR23 questionnaires was conducted between May and June 2024 with patients diagnosed with breast cancer posting in the Inspire patient online forums.

b Not applicable.

## Discussion

The goal of this feasibility study is to investigate whether we can reliably gain QoL information from posts of patients in online communities. To this end, we have introduced a new methodology that examines the content of posts in relation to data from QoL surveys completed by the same cohort of patients whose posts we have access to. This has allowed us to not only assess how frequently users post information that is relevant to QoL, but also measure the degree to which this information corresponds to the answers to the corresponding questions in the questionnaires. This methodology is novel, as it has not previously been applied to analyzing whether information shared by patients online can reliably estimate individual QoL data. We have provided evidence that this methodology is sound, showing that the information can be coded at good levels of agreement and that there is some objective “ground truth” in the information shared by patients online that can be consistently interpreted as providing answers to the survey questions. With regard to RQ1, we can say that patient online forum posts indeed contain QoL-related information. The questions most frequently addressed in those posts (RQ2) are “Did you worry?” “Did you feel tense?” and “Have you had pain?” Moreover, the online posts align well with the actual QoL questionnaire responses (RQ3), achieving an *F*_1_-score of 0.7, with even better results when grouping similar questions (*F*_1_-score =0.8 for fine-grained and 0.9 for coarse-grained grouping).

In particular, Fleiss κ values were nonnegative with “Did you have headaches?” achieving the highest score of 1.0. Only 6 out of 53 questions showed agreement values of 0, with the mean Fleiss κ value being 0.5 (SD 0.28), indicating moderate to solid agreement among the annotators, especially considering the complexity of the task.

We have further quantified the degree to which patients share information that can be mapped to questions from the EORTC QLQ-C30 and QLQ-BR23 questionnaires.

From the total of 2669 annotated posts and comments, 613 (23%) answered at least 1 question from the used EORTC questionnaire. For the last 6 months (since the end of the survey), even 217 out of 580 posts (37.4%) contain QoL-relevant information. As these numbers might be affected by selection bias (indicated by older posts containing less QoL-relevant information), they should be seen more as upper bounds compared to scenarios where the data of an arbitrary patient are investigated.

We have further shown that there is a substantial correlation between the information that participants have provided in their online posts and the survey responses. We have proposed to rely on standard evaluation metrics as used in machine-learning research to estimate the correctness of the information provided by participants in their online posts compared to their answers to corresponding questions in the questionnaires.

The average score for the most recent 6 months of data across all 33 questionnaire questions that had answers in the respective data was an *F*_1_-score of 0.7, indicating that posts and comments containing QoL-relevant information can reliably predict questionnaire answers. Similarly, from the patient level, an average of 63.4% (SD 33.5%) of the estimated answers to the questions is correct. This indicates a high predictive power of forum posts which contain QoL information.

When grouping similar questions together, we even get a mean *F*_1_-score of 0.8 (SD 0.154) for fine-grained grouping and a mean *F*_1_-score of 0.9 (SD 0.01) for coarse-grained grouping when applying the time deviation filtering, excluding posts and comments talking about events and experiences from an unspecified or distant past. Without filtering, we get mean *F*_1_-scores of 0.7 (SD 0.353) without grouping, 0.8 (0.158) with fine-grained grouping, and 0.9 (SD 0.051) with coarse-grained grouping.

The most frequently answered questions (RQ2) include “Did you worry?,” “Did you feel tense?,” “Have you had pain?,” “Were you limited in doing either your work or other daily activities?,” “Were you tired?,” “Have you felt nauseated?,” “Were you worried about your health in the future?,” “Have you lacked appetite?,” and “Did you feel depressed?” These questions are not particularly specific to breast cancer, thus indicating patients typically discuss their QoL online more on a general level or in such a specialized manner that none of the EORTC questions fit well as shown by the high number of “Did you feel ill or unwell?” occurrences.

One inherent problem of the collection and analysis of the questionnaire data is related to missing data, as often patients do not fill out all information. In our case, the rate of completeness for the survey data was 100%, as the completion of all questionnaire items was required. Two questions of the form “If you were/had …” allowed choosing “no answer,” which, however, we do not consider missing data but instead a different way to answer the question.

However, not all questions were answered in posts of all patients. For patients who have shared QoL-relevant information in their posts, only about 10.8% (SD 9.8%) of the questions could be answered by information given in the posts. This means that the available data are typically not sufficient to answer all questions of the EORTC QoL questionnaire, but at least some parts can be reliably extracted for each patient. We also investigated ways to deal with data sparsity by grouping questions and considering longer time periods, which help to get more insights into the QoL without losing too much reliability.

Overall, our results show that users of online communities, such as Inspire, indeed post information about their QoL, providing important information that can be consistently and objectively mapped to the categories of standard questionnaires.

Our results have important implications. First, the results suggest that we could potentially seek answers to questions that are typically not included in standardized QoL questionnaires. Furthermore, there are nuances in the posts of patients that are not directly captured by the questionnaires. On one hand, the topics that patients do or do not bring up in online discussions may indicate which topics are currently the most important for that patient, potentially referring to specific aspects of the relatively broad areas covered by the QoL questionnaires. On the other hand, this can provide insights into areas, which are not covered by the QLQ because they are unique to that patient (eg, due to certain living circumstances). This hints at the potential of our methodology to complement data obtained from standard QoL questionnaires.

### Comparison With Prior Work

In recent years, there have been numerous studies investigating whether QoL-related information can be gained from unstructured data sources. However, our work differs from related approaches in substantial ways.

For one, our approach leverages different types of data considered. We considered social media posts from dedicated health care online communities or more precisely the breast cancer forum of Inspire. Other work has focused on clinical reports [30,31] or health records [31,32], which, in spite of also having unstructured elements, considerably differ in terms of content and style from social media data. Among similar approaches considering social media, many approaches have sourced Twitter data [13,15,33-37], in contrast to the specific health care online community data that we used. An exception is the work by Leung et al [38,39] who have used data from cancer support groups. However, their data originate from chats moderated by health care professionals instead of posts from organic, forum-like online communities. Their study had a narrower focus by studying signs of emotional distress in patients participating in the online discussions instead of the broader focus of capturing QoL-related information in our study. An overview of how social media contents can be analyzed for health care is given by Fu et al [40].

Our study has aimed to gain insights into the QoL of individual patients based on their posts in health care communities. In contrast, the goal of many other studies is to discover aggregate trends for a certain geographic segment of the target population [13,15,36]. Other studies are interested in population-wide trends and effects related to how a disease, intervention, or treatment affects patients in general, instead of estimating values for specific users [9,11,12,37,41-52]. Such population-wide analyses can support the creation of conceptual models of a disease [9,42], whereas other studies have focused on general and individual health [33], recognition of discussions about health services [53], or emotional distress in participants of online chats [38,39].

Some studies have been conducted in relation to existing QoL questionnaires. For example, Tapi Nzali et al [54] have also used the EORTC QLQ-C30 and QLQ-BR23 questionnaires, aiming at identifying general topics that patients diagnosed with breast cancer discuss in relation to EORTC questionnaires. Gries and Fastenau [52] have relied on items in the EORTC QLQ-C30 and QLQ-MY20 questionnaires to investigate concepts of interest, symptoms, and impacts discussed by patients with multiple myeloma on a specific health care social media platform. These studies analyze general trends rather than predicting QoL for individual patients.

Renner et al [55] annotated data from 19 health-related forums using the Short Form Health Survey SF-36 [56] and Euro Quality of Life 5 Dimensions [57], but they did not compare and contrast survey responses with the information in patient posts as we do.

There are few studies that have analyzed the information posted by patients in relation to ground truth QoL data as available in the existing QoL questionnaire data. For example, Zivanovic et al [15] have compared Twitter data originating from the city of Bristol with official QoL data for this city. They analyzed the data on an aggregate level for the whole city rather than for individual patients as we do. Twitter has also been used by Sarma et al [35] who compared information obtained from Tweets with ground truth data of Centers for Disease Control Healthy Days Questionnaire [58]. The questionnaire used is much shorter than the one used in our study, providing a less detailed view on patients’ QoL. Cotté et al [16] have compared data from cancer-specific forums with data obtained from the Functional Assessment of Cancer Therapy-General [59] and EORTC QLQ-C30 questionnaires. Their main focus has been on investigating the impact of immune checkpoint inhibitors, but not investigating the QoL impact for individual users.

Although this work focuses on breast cancer, related work covers a wide range of diseases. These diseases include Parkinson disease [9,10], amblyopia [41], presbyopia [42,49], cancer [16,30,48,51], dry eye disease [43], chronic ocular pain [44], Sjögren disease [11], chronic obstructive pulmonary disease [12], systemic lupus erythematosus [45,60], immunoglobulin A nephropathy [46], complement 3 glomerulopathy [47], atopic dermatitis [50,61], obesity [36], depression [62], and (general) mental health [13,14].

In summary, our work advances the field by paving the way for a new option to monitor patients’ QoL. As elaborated in the previous section, analyzing forum posts with respect to QoL questions can be used to complement existing instruments for assessing QoL, helping to focus on the most relevant aspects of patients’ QoL as well as to discover areas potentially not covered by existing instruments in detail. Additionally, possible ways to automate the process of identifying QoL-relevant information in online posts using, for example, artificial intelligence methods, pose an interesting path of future research.

### Limitations

Despite carefully annotating the data and collecting ground truth data through a survey, our work has multiple limitations, which are briefly discussed in this section.

In our study, the investigated population has been limited to those active on the Inspire platform. However, many other online patient communities exist, and both the frequency of QoL-relevant utterances as well as their predictive power might be different.

Similarly, the fact that older posts in our dataset discuss QoL-relevant topics less often indicates that selection bias might be present in our data, as patients currently interested in QoL might be more likely to have participated in our study. Thus, our results have to be considered more like an upper bound to the possible insights about patients’ QoL from posts in online forums.

Moreover, we also found signs of recall bias in the data. For example, we found that some patients talked about how much they worried about different topics shortly before participating in the study, but then answered that they did not worry at all. The occurrence of such phenomena has not been systematically investigated in this work and might thus have an impact on the results.

Certain highly intimate topics, such as aspects related to sexuality or body image, were rarely discussed in the forum posts. Given the public nature of the data, which is to be expected, however, it represents a limitation in terms of depth and scope of insights that can be obtained from such a dataset. Compared to one-on-one interviews, forum data may provide a less comprehensive picture of patients’ concerns in these areas.

Finally, data sparsity and unequal distribution of posts and comments among forum users are typical problems when considering social media. As illustrated by the long-tail distribution in Figure 2, there are a few users with many posts and comments in our dataset, but the majority of users have much fewer posts available. The degree to which we can obtain QoL information is thus dependent on the posting behavior of a particular user. Statistics and numbers presented in this work thus have to be interpreted as upper bounds for the potential to extract QoL information from a randomly chosen user of the Inspire communities. Similarly, due to the general data sparsity, labels that are scarcely covered by our data sample might be present more prominently for other users, which have not been part of our study, and analogously, labels with many occurrences in our data might be more rare to find for other users.

### Conclusions

This study has investigated the feasibility of gaining insights about QoL from posts of patients in online forums. Toward this goal, we have recruited patients diagnosed with breast cancer from Inspire. These patients have filled the EORTC Quality of Life Questionnaire and allowed us to analyze their posts and comments in relation to their answers given in the questionnaire. By this, we have been able to compare the information provided in their posts with the answers provided in the questionnaires. Our key findings are that between 23% and 37.4% of patients’ posts contain information that can be mapped to at least one of the questions of the questionnaire. Furthermore, we could show that the QoL information in posts is reliable, corresponding to a large extent to the answers given in the questionnaire. Our findings thus show that the information shared by patients in online posts can contribute to a better understanding of how the disease affects their QoL.

While the information provided by patients in posts shared in online forums cannot replace the collection of data via questionnaires, it represents a low-cost, low-burden alternative to collect insights about QoL effectively. Most importantly, the information in posts can help us to discover aspects related to disease burden and QoL that are not captured by existing questionnaires. This could even provide guidance on how survey instruments could be extended to cover those aspects that are most burdensome to patients.

Future work should investigate how exactly the information provided by patients online can complement existing QoL assessment methodologies. A further line of research relates to how the information can be extracted automatically, in order to avoid having to scan and read thousands of posts. Given the current state of research on information extraction, this seems to be a promising avenue to investigate. Additionally, first experiments conducted in this direction yielded promising results.

All in all, our work contributes to the foundation for a novel low-burden alternative for monitoring patients’ QoL and complementing existing QoL instruments. Moreover, this opens up interesting new avenues of research on how to automate the process including using artificial intelligence methods and thus further lowering the burden for both patients and clinical staff.

## Supplementary material

10.2196/76044 Multimedia Appendix 1 Annotation guidelines and insights from annotation.

10.2196/76044 Multimedia Appendix 2 Distribution of annotations between labeled questions.

10.2196/76044 Checklist 1 CHERRIES checklist.

## Abbreviations

CHERRIES: Checklist for Reporting Results of Internet E-Surveys
EORTC: European Organisation for Research and Treatment of Cancer
FN: false negatives
FP: false positives
QoL: quality of life
TN: true negatives
TP: true positives
