# Supplementary material for: Extracting Quality of Life Information of Patients Diagnosed With Breast Cancer From Health Care Online Forum Posts: Data Feasibility Study
Source: JMIR Cancer. 2026 Apr 30;12:e76044. doi: 10.2196/76044 (PMC13132019; doi:10.2196/76044)
Supplement: Multimedia Appendix 1 [file cancer-v12-e76044-s001.docx]

# Appendix

Annotation Guidelines

Annotating Online Forum Posts Regarding Quality of Life Questions

Version 1.2

1.7.2024

David M. Schmidt

Antonia Frank

Raoul Schubert

# Introduction

This document aims for guiding annotators in annotation online forum and social media posts w.r.t. the question which quality of life questions from the questionnaires EORTC QLQ-C30 and EORTC QLQ-BR23 are answered or at least partially discussed in them. The EORTC QLQ-C30 contains 30 questions and the EORTC QLQ-BR23 23, respectively. Those questions as well as two special labels used during annotation are described in the following.

# General Annotation Setting

Each post is annotated separately on sentence level in general, i.e. inside a post, text spans mark the parts of a post which answer the corresponding quality of life questions. Those text spans always comprise one or more full sentences, i.e. annotation of parts of a single word are not possible. Marking parts of a sentence on word-level is possible if two or more parts of a sentence can be clearly assigned to different labels, e.g. the enumeration “I'm always tired, forgetful, weight gain, hair loss, headaches, depression and joints aches.” allows to clearly assign different labels to the enumerated symptoms. In that case each clearly identifiable unit is annotated with the corresponding label instead of annotating the whole sentence with all enumerated labels. Whenever the distinction is not clear, annotating the whole sentence with all relevant labels is preferable.

Except for the special labels “Exact Match” and “Negative”, every part of a post which (maybe even just remotely) answers or touches one of the following quality of life questions is marked and annotated with the corresponding label. In cases that answer a question especially well/precise, the “Exact Match” label is *additionally* assigned to the same text span in order to allow differentiating which label the exact match is related to. Assigning “Exact Match” only exactly to spans with questions that are exactly answered is especially important if multiple labels are assigned to overlapping text spans. In that case only the text span of the exact match gets the “Exact Match” label and no new text span is created for the exact match in order to allow the assignment to the correct label later. For questions that are negatively formulated, e.g. “I do not have any pain at all”, the “Negative” label is *additionally* assigned to the label of the corresponding quality of life question.

Any utterance (partially) answering one or more quality of life questions is annotated. This also means aspects like (but not limited to) the following are not considered during annotation:

- Time of the described events, e.g. when symptoms happened, no matter whether it was multiple years or just days ago
- Whether the described events are results of, e.g., a previous cancer treatment or not
- Patients talking about other people, i.e. “My sister has …” are annotated as if the patients talk about themselves, but a comment with the corresponding post ID is made noting the potentially different reference of the corresponding statements to allow later exclusion of said statements if necessary.
- Utterances implicitly indicating an answer to one of the quality of life questions are only annotated in obvious cases, excluding for example pain medications indicating that the patient is having pain etc. or other circumstances that suggest an answer to one of the questions but are not stated explicitly.
- Potential ambiguities are annotated in a very direct literal reading, leaving a comment with the post ID for later discussion or exclusion if necessary

# Categorization of EORTC QLQ Questions

The following examples are fictional but close to actual posts encountered during annotation.

## Global health status/QoL

- How would you rate your overall health during the past week?
- How would you rate your overall quality of life during the past week?

## Functional

- Physical functioning
  - Do you have any trouble doing strenuous activities, like carrying a heavy shopping bag or a suitcase?
  - Do you have any trouble taking a long walk?
  - Do you have any trouble taking a short walk outside of the house?
    - Example: “To treat my thyroid cancer, about 15 years ago, I underwent radiation therapy after surgery. Roughly five years ago, doctors noticed I was developing pulmonary fibrosis, likely as a long-term effect of the radiation. Now I find myself easily short of breath even when I take short walks around my neighborhood. Next week, I have an appointment with my pulmonologist, who might recommend a CT scan to assess how things are progressing.”
  - Do you need to stay in bed or a chair during the day?
  - Do you need help with eating, dressing, washing yourself or using the toilet?
- Role functioning
  - Were you limited in doing either your work or other daily activities?
  - Were you limited in pursuing your hobbies or other leisure time activities?
    - Example: “I understand the challenges you’re facing and the effort it takes each day to manage everything. Before my diagnosis, I was constantly on the go—hiking, baking, tending to my plants, and always keeping busy around the house. I might even have some of the same symptoms you do, as we have similar conditions. I also went through radiation—20 sessions, since I chose to avoid chemotherapy due to another health issue. Now, several years later, I still deal with limitations I didn’t expect. My doctors sometimes dismiss my concerns, attributing them to age, which feels frustrating. But my mind is sharp; I haven’t lost my mental clarity or problem-solving skills.

It’s so comforting to know I’m not alone, especially when stress flares up my anxiety. I’d never experienced anxiety before, not even during my initial treatment. These days, I rest when I’m worn out and in pain. There’s no choice but to pause—I simply can’t push through like I used to. The fatigue hits quickly after doing just a little too much.

Some of my favorite activities, like painting and knitting, are harder now, which is frustrating. I take things slower and break up tasks. Connecting with close friends has been a huge help; my friend of over 40 years is always there to listen and reassure me. Staying positive and finding reasons to be grateful has become part of my routine, as has advocating for myself in appointments. I also see a holistic doctor now, who helps with supplements that boost my energy and immune system.”

- Emotional functioning
  - Did you feel tense?
    - Very similar to “Did you worry?”, but nevertheless one should not annotate both at the same time but instead make a decision based on the tendency which label fits better in the given situation. If the inter-annotator agreement is too low for the annotated data, the labels can be merged later.
    - Used to annotate fear and concerns
  - Did you worry?
    - Very similar to “Did you feel tense?”, but nevertheless one should not annotate both at the same time but instead make a decision based on the tendency which label fits better in the given situation. If the inter-annotator agreement is too low for the annotated data, the labels can be merged later.
    - Used to annotate fear and concerns
    - Example: “Thank you for sharing your experience. I recently saw a naturopathic specialist who suggested a list of supplements and a prescription, even though I didn’t feel particularly unwell. I think gut health is important, but I’m hesitant to see it as the answer for everything. I’m holding off until I get more clarity from my tests. The worry has really been taking its toll on me, and with the treatment meds, my options are limited due to potential side effects. It’s a struggle. This past week was especially tough with some unexpected flare-ups.”
  - Did you feel irritable?
    - Used to annotate anger and frustration
  - Did you feel depressed?
    - Example: “I was hesitant to try any new medications. My system reacts strongly to them, and I often deal with feelings of depression and anxiety. We were finally in a good place with things. But each person has such a unique experience.”
- Cognitive functioning
  - Have you had difficulty in concentrating on things, like reading a newspaper or watching television?
    - Example: “In June of 2018, I went through a lumpectomy followed by the same type of chemo your sister is undergoing. The first round was definitely the hardest. Stretching exercises helped a bit with the muscle pain, though I still had persistent aches in my arms and back during and after treatment. The exhaustion improved slightly over time, but I still struggle with it now and then. One thing I found especially hard was concentrating—it made things like reading a book or following a TV show really frustrating. I also lost my hair, most of my nails, and even my sense of taste for a while. Eating became a challenge because everything had this strange bitter flavor.“
  - Have you had difficulty remembering things?
    - Example: “My treatment was extremely hard on me. I experienced severe side effects like allergic reactions to medications, extreme fatigue, digestive issues, confusion, dizziness, and difficulty recalling dates. It’s been over a year since I completed my treatment, and I’m still working on feeling like myself again. Everyone’s journey is different.”
- Social functioning
  - Has your physical condition or medical treatment interfered with your family life?
    - Example: “I was diagnosed with Stage III Triple-Negative Breast Cancer in early 2015. For the past six months, I’ve been grappling with this diagnosis and the profound changes it has brought to my life. I work as an art teacher at a community college and am also an amateur classical guitarist, performing occasionally at local events. I’m 49 years old, a mother of two grown sons, and the oldest of three siblings. My dad, who is 81, has been living with me since his stroke two years ago. Currently, I’m undergoing treatment with Carboplatin and Taxol, which has left me exhausted but still determined to keep teaching part-time. The hardest part for me has been balancing my health and my role in the family. Before my diagnosis, I handled most of my dad’s care—managing his medications, doctor’s appointments, and mobility challenges. He’s largely dependent on me, but I’ve had to hire a part-time caregiver because I can’t physically do everything I used to. Dad tries to be supportive and insists on attending my appointments when he can, but I know he struggles with the shift in roles. It’s hard to watch him worry so much about me now.”
  - Has your physical condition or medical treatment interfered with your social activities?
- Body image
  - Have you felt physically less attractive as a result of your disease or treatment?
    - “Has anyone else experienced drastic changes in their appearance after treatment? I used to have such healthy, glowing skin and a confident smile, but within a few months of finishing treatment, everything seemed to change. My skin now looks pale and dull, my hair has become thin and brittle, and I’ve started noticing deep wrinkles that weren’t there before. On top of that, my teeth have become weak—they chip so easily now, and I’m terrified they’ll get worse. It’s hard to even look in the mirror sometimes. I feel like a completely different person, and it’s taken such a toll on my self-esteem. My friends and family have noticed too, and while they’re supportive, I can’t shake the anxiety and sadness from these changes. I know being alive is what matters most, but adjusting to such a transformation feels overwhelming and isolating.
  - Have you been feeling less feminine as a result of your disease or treatment?
  - Did you find it difficult to look at yourself naked?
  - Have you been dissatisfied with your body?
    - Example: “I’ve been debating whether to continue on my current medication for another few years. It put me into early menopause, and ever since, I’ve felt so disconnected from my body. I barely recognize myself anymore—both physically and mentally. Could it be the treatment causing these changes, or is this just how menopause affects me? I’d really appreciate advice from anyone who’s been through this.
- Sexual functioning
  - To what extent were you interested in sex?
  - To what extent were you sexually active? (with or without intercourse)
- Sexual enjoyment
  - To what extent was sex enjoyable for you?
- Future perspective
  - Were you worried about your health in the future?
    - Example: “It's been two years since my diagnosis. Physically, I'm doing okay now. The tingling and numbness in my hands have lessened significantly since I started using compression gloves regularly. My hair has grown back, though it's a completely different texture and color than before. Emotionally, it's a rollercoaster—I still have moments where I break down, especially when I think about what the future might hold for my health. But I'm slowly coming to terms with this "new normal." It's tough to redefine who I am, but I'm trying to embrace the version of me that's emerging. Sometimes I miss the carefree, uninformed person I used to be, but in some ways, I feel stronger now.”

## Symptoms

- Fatigue
  - Did you need to rest?
  - Have you felt weak?
    - Example: “Wishing you all a peaceful and healthy start to the new year. I was diagnosed with Stage 1B Invasive Lobular Carcinoma, Hormone Receptor + and HER2 -, back in April. I completed 25 sessions of targeted radiation therapy and tried Letrozole for about two weeks before stopping due to severe side effects. About a month later, I started experiencing strange symptoms—weakness, heaviness, burning sensations in my muscles, joint stiffness, fatigue, and occasional difficulty walking or climbing stairs. My hands sometimes feel useless, and the insomnia and anxiety have been overwhelming.This has been ongoing every day, and it feels like things are slowly worsening. I’ve been referred to a neurologist and finally have a rheumatology consult scheduled for April, but everything feels like it’s moving at a snail’s pace. So far, I’ve had several blood panels done, nerve conduction studies, and an MRI scheduled for next week. Has anyone else gone through something similar? One theory is that my cancer treatment triggered an immune response that's now causing my body to attack itself.
  - Were you tired?
    - Example: “I felt completely drained after my chemo sessions, barely managing to make it back home to rest each time. Once the sessions ended, the exhaustion started to ease, but I still find myself feeling fatigued on most days. The healing balm I first tried didn’t help much and caused some irritation, but switching to a chamomile-based cream and cooling gel worked wonders for the discomfort and irritation. I’m glad you’ve found some answers, and I hope your current treatment helps you recover soon. Wishing you the best, take care!”
- Nausea and vomiting
  - Have you felt nauseated?
    - Example: “I was diagnosed with Stage 2 Lobular Carcinoma. I had a double mastectomy followed by chemotherapy and targeted radiation therapy. Right now, I’m taking Orinza twice daily and Femrex once a day, along with monthly Zeprel injections. My body is struggling to adjust to Orinza—it’s been about a month since I started, and I’m dealing with constant nausea and frequent stomach upset. The nausea lasts for hours, and I can’t seem to get it under control. I’ve tried anti-nausea medication, but it only helps a little. Has anyone else gone through this with Orinza or a similar treatment? Did you find anything that made the side effects more manageable? I’d appreciate any advice!”
  - Have you vomited?
    - Example: “They say what doesn’t break us makes us stronger. Back when I started treatment years ago, I remember being so sick from the chemo, I couldn’t keep anything down for days. It felt unbearable. Over time, though, I was prescribed newer medications to manage the side effects, and it became a lot more manageable. Even though that first chemo cycle was the hardest thing I’ve ever gone through, I know I wouldn’t still be here without it.”
- Pain
  - Have you had pain?
    - Example: “I received my third round of treatment a few days ago. The effects of the first round are still lingering, and it's becoming increasingly difficult to deal with both the physical and emotional pain. The fourth round will be my final one, but I haven't yet decided what path I will choose after that.”
  - Did pain interfere with your daily activities?
- Dyspnoea
  - Were you short of breath?
- Insomnia
  - Have you had trouble sleeping?
- Appetite loss
  - Have you lacked appetite?
    - Example: “Hello all, just had surgery followed by two bowel issues, and started my first round of chemotherapy two days ago. I'm dealing with Stage 3 ovarian cancer that has spread to my bones. After the surgery, I noticed I just couldn't bring myself to eat, and even now I really have to push myself to eat anything. I've been trying shakes to help with keeping up my strength and nutrition. Has anyone gone through something like this? Could it be the stress from everything going on, or maybe the bowel problems have just made food unappealing?”
- Constipation
  - Have you been constipated?
    - Example: “I never thought of that since I don't eat stuff like that for breakfast. And I do need fiber since my medication gives me pretty bad constipation.”
- Diarrhea
  - Have you had diarrhea?
    - Example: “It's been about a month, and I’ve been dealing with persistent stomach issues, including frequent diarrhea and nausea. The diarrhea is particularly troubling, as it lasts most of the day, and I haven't found much relief. I’ve tried over-the-counter remedies like Pepto-Bismol, but no real improvement. My doctor has also prescribed additional medication to manage the symptoms, but it’s still a struggle. I’m wondering if anyone else on a similar treatment has experienced these side effects? Any tips or advice would be greatly appreciated.”
- Financial difficulties
  - Has your physical condition or medical treatment caused you financial difficulties?
    - Example: “Hi everyone! I’ve been dealing with some major insurance issues lately and thought I’d reach out to see if anyone else is going through something similar. I recently moved to a new city and have been trying to sort out my healthcare coverage ever since. It’s been a real hassle, and I’m still trying to get things figured out. While it hasn’t completely interrupted my treatment, the costs for medications have been piling up. Just wanted to vent a bit, in case anyone else has had a similar experience.“
- Systemic therapy side effects
  - Did you have a dry mouth?
    - Example: “Has anyone had experience with the side effects of "Prevemast"? My meds have caused enough issues, sleeplessness and a dry mouth that made it hard to function. I’m also looking for ways to boost my energy and raise my blood counts, as I really don’t want to keep having transfusions every other week.”
  - Did food and drink taste different than usual?
    - Example: “I have been on my new medication for a short while.. For the past few weeks I have effectively not been able to taste ANYTHING. Did anybody else lose their sense of taste after getting diagnosed?”
  - Were your eyes painful, irritated or watery?
    - Example: “I have been on anastro for years and have been plagued with irritated eyes and blurry vision. Hope you’ll get the treatment you need without any side effects!”
  - Have you lost any hair?
    - Example: “I cant believe it, lost all my hair! it started coming back around month 11. Now I am on the 12th dose. Had a friend who did cold cap and he had hair thinning. So its not necessarily a sure thing you lose all your hair on this drug.
  - Did you feel ill or unwell?
    - Very broad and general question and therefore used as a “catchall”/superclass question of no more specific symptom question matches, in those cases always without “Exact Match”.
    - Also used to label general negative statements such as “I feel well”, then together with “Negative”
    - If an utterance matches closely with the question, i.e. “I feel ill/sick” or some other undefined physical unwellness is expressed, the label is used in conjunction with “Exact Match” to emphasize the question itself is answered and not just some not further specified symptom that matches none of the other labels.
  - Did you have hot flushes?
    - Example: “I have had pretty bad hot flushes to be honest. Also I now have osteopenia, so I get an infusion every six months to build more bone.
  - Did you have headaches?
    - Example: “I’ve been on the drug for quite a while at this point. Daily headaches and other pains are pretty common for me throughout the day. I took a vacation from it while I was undergoing another unrelated surgery and during the first few weeks post surgery I felt so much better! Even had more energy!
- Breast-related symptoms
  - Have you had any pain in the area of your affected breast?
    - Example: “I recently had a lumpectomy, radiotherapy and now on the meds for the first time. Just a weeks ago I started feeling pain, redness and also irritation under one of my breasts. Did any of you have skin that looked red and like it’s coming off? Which physician did you see and what was therecommended treatment like?”
  - Was the area of your affected breast swollen?
  - Was the area of your affected breast oversensitive?
  - Have you had skin problems on or in the area of your affected breast (e.g., itchy, dry, flaky)?
    - Example: “ I am considering going back to see the oncologist. The area seems more like after a burn than after an infection. I do have pain and aching. Did any of you have skin that looked peeled?
- Arm symptoms
  - Did you have any pain in your arm or shoulder?
    - Example: “When it comes to my arm I have joint and muscle pain! I will be 83 soon and hopefully can stop seeing all the doctors! I see them almost weekly by now for one reason or another!. I wish you all the best!.”
  - Did you have a swollen arm or hand?
    - Example: “I had a mastectomy and now my left arm is hugely swollen and the hand is so swollen that I have stretch marks starting to form!. Please help, if you have found something that helps! Thank you so much!”
  - Was it difficult to raise your arm or to move it sideways?
    - Example: “I was told the biopsy showed NED and the doc thought he got a good sample but the excrutiating pain under my arm is just unbearable to the point I can't even raise my arm! I wrote to the doctor and she advised me to see her as soon as the bruising goes down.
- Upset by hair loss
  - Were you upset by the loss of your hair?
    - Example: “Hi everybody. The results came back today, and unfortunately, the cancer is both ER and PR positive. The physicianrecommends chemotherapy and radiation, and he's optimistic about the treatment plan. I’m just devastated about the thought of losing my hair AGAIN and the pain that chemotherapy might bring. I’m also worried about the possibility of recurrence.”

# Special Labels

### The “Exact Match” Label

If a text span answers one of the quality of life questions in such a way that even the corresponding questionnaire answer (from “Not at all” to “Very much”) is reasonably clear, the same text span is *additionally* labeled with the “Exact match” label and assigned to the identical text span that the corresponding question label is assigned to. Other not exactly matching questions then have to be assigned to a different text span with potentially identical content.

### The “Negative” Label

If a text span unambiguously describes an event in a negative way, e.g. “I did not have pain at all”, instead of in a positive way, e.g. “I did have pain”, the corresponding text span is *additionally* labeled with the “Negative” label, indicating that this text span suggests an answer like “Not at all” or similar to the corresponding quality of life question. The direction is always meant in the way the question is asked and corresponding to the “Not at all” or “A little” answers.

Insights from Annotation

The annotation process uncovered both opportunities and challenges in mapping user-generated healthcare posts to QoL questions. The decision was made to exclude posts that described the conditions of individuals other than the posters themselves, such as relatives or friends. While such posts often contained health-related information, they were not directly relevant to the poster’s personal QoL and thus were excluded to maintain focus on the primary objective of the study, focusing on the authors of the posts exclusively. Similarly, we made the methodological choice not to equate mentions of illnesses or medications with their symptoms. For example, the mention of neuropathy or a specific pain medication was not automatically labeled as “pain”. This decision was made to avoid overgeneralization and to maintain fidelity to the exact language used, rather than to rely on the interpretation of implicit meanings in the posts.

Applying the “ill/unwell” label posed particular challenges as well. Symptoms such as low blood pressure, fever, or a “weepy nose” often did not fit cleanly into the predefined categories but were the closest matches available. This suggests the need for more nuanced labels or subcategories that can differentiate between specific symptoms and general states of wellness. Additionally, ambiguous language in posts often required interpretation on a case-by-case basis. Colloquial expressions, such as “hand-foot thing” for instance, were particularly difficult to annotate without additional context. In this case, it was likely a reference to hand-foot syndrome, but the lack of explicit confirmation in some posts required the annotators to make educated guesses. Similarly, words such as “fatigue” often had multiple potential meanings, ranging from physical exhaustion to emotional demoralization, complicating the labeling process which we chose to discuss on individual bases. Another challenge that emerged during the annotation process was the potential overlap between certain questionnaire items, particularly “Did you worry?” and “Did you feel tense?”. These two labels often addressed similar emotional states, making it difficult to draw clear boundaries between them based on user posts where the words *worry* and/or *tense* are not explicitly mentioned. As such expressions of general unease or anxiety could plausibly align with either question, depending on the specific phrasing or context.

Contextual dependencies added another layer of complexity. Many posts included anaphoric references, such as “this” or “it,” referring to previously mentioned symptoms or conditions. Annotating such references required considering preceding sentences to determine the referent, which means sentence boundaries had to be crossed for the annotation in order to prevent loss of meaning when encountering these deictic expressions. Another challenge arose from the diversity of conditions described in the posts. As some symptoms appeared to result from issues such as side effects from COVID-19 vaccinations, rather than the cancer treatments themselves, we have ultimately chosen to take these symptoms into account since they are not easily distinguishable and can still be considered important data points for assessing QoL.

The dataset itself presented limitations that impacted the insights derived from the annotations. Sensitive topics, such as sexual activity or body image, were rarely discussed, likely due to the public nature of the forum. This highlights the limitations of community-generated data compared to private, structured clinical assessments. Furthermore, the self-reported nature of the posts introduces potential recall bias, as users may not consistently describe their experiences in detail, may describe past issues in forum posts and then not mention them on the questionnaires themselves or focus on specific aspects of their health. Selection bias was also a concern, as the dataset was limited to active forum participants, who are likely overrepresented among individuals particularly interested in discussing QoL topics. As such, the data may better reflect an upper bound of engagement with QoL issues rather than the average experience of breast cancer patients. Supporting this interpretation, the proportion of relevant posts decreases with temporal distance from the survey period, which suggests that discussions tend to focus on QoL-related themes when participants are actively engaged with the topic.

Despite these challenges, the annotation process revealed that a significant portion of the posts contained information relevant to QoL questions, demonstrating the potential of online forum data for assessing patient experiences. However, it also showcased the importance of refining annotation schemes beyond conventional QoL questionnaires, addressing ambiguities and acknowledging the limitations of this data source to enhance the validity and reliability of future analyses.
