# Supplementary material for: Extracting Quality of Life Information of Patients Diagnosed With Breast Cancer From Health Care Online Forum Posts: Data Feasibility Study
Source: JMIR Cancer. 2026 Apr 30;12:e76044. doi: 10.2196/76044 (PMC13132019; doi:10.2196/76044)

Figure 1: Distribution of annotations between labeled questions. The number on the horizontal axis is the number of the corresponding question in the EORTC QLQ-C30 and QLQ-BR23 questionnaires. 54 is assigned to the special additional label “Exact match” and 55 to “negative.” The online survey using the EORTC QLQ-C30 and QLQ-BR23 questionnaires was conducted between May and June 2024 with patients diagnosed with patients with breast cancer posting in the Inspire patient online forums. EORTC: European Organisation for Research and Treatment of Cancer.
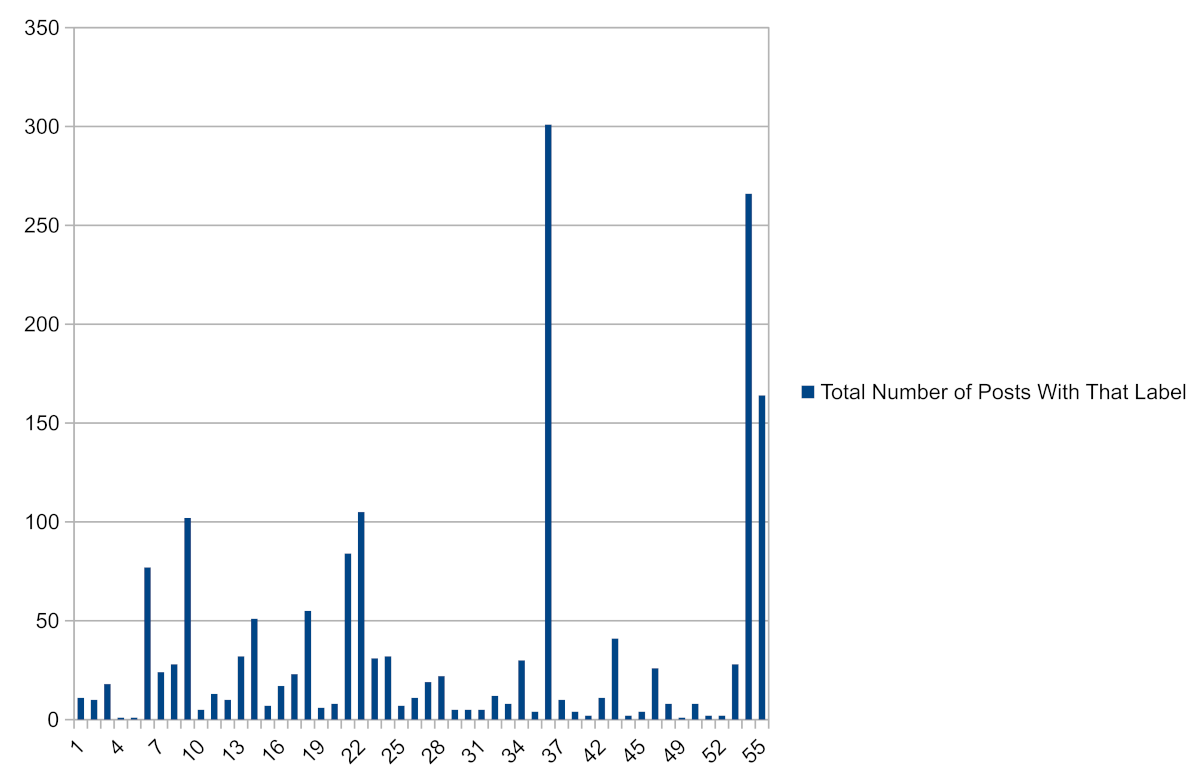

Supplement: Multimedia Appendix 2 [file cancer-v12-e76044-s002.docx]
