# Supplementary material for: Extracting Quality of Life Information of Patients Diagnosed With Breast Cancer From Health Care Online Forum Posts: Data Feasibility Study
Source: JMIR Cancer. 2026 Apr 30;12:e76044. doi: 10.2196/76044 (PMC13132019; doi:10.2196/76044)
Supplement: Checklist 1 [file cancer-v12-e76044-s003.pdf]

Checklist Item	Explanation
Describe survey design	Participants were recruited from the “Breast Cancer” and “Advanced Breast Cancer” communities on Inspire.com. Eligibility criteria included:<br>- Female breast cancer diagnosis<br>- Age 18 years or older<br>- Residency in the United States<br>- At least one post or comment in the respective Inspire.com communities<br>Residency in the United States was included due to Inspire.com being based in the United States. All eligible community members were invited to participate (i.e., Convenience Sampling), with the first 100 respondents receiving a $20 gift card.
IRB approval	Our study has been approved by the Ethics Committee of Bielefeld University under application No. 2023-216-W1.
Informed consent	Informed consent was obtained from all participants, granting permission to analyze their posts and comments for this study. Consent text: “The goal of the study is to understand whether we can extract information about the quality of life of breast cancer patients from their posts in the Inspire community. For this purpose, this study will collect answers from Inspire members on their quality of life using standard instruments, European Organization for Research and Treatment of Cancer (EORTC) questionnaires in particular. The answers to these questionnaires will then be correlated with the information provided by study participants in their forum posts, in order to understand whether the information provided is rich enough to derive quality of life assessment from them. If successful, the data will be used to train an AI system to extract the quality of life information from the forum posts automatically as a supplementary, but validated method of capturing additional real-world quality of life data to subsequently improve patient care.<br>Remember, your answers are anonymous and only be reported in aggregate along with others participating in the research.”
Data protection	Personal information (like questionnaire answers) are stored in an encrypted form only and only on local computers of the respective institutions.
Development and testing	To compare patient-reported QoL in online posts with structured assessments, we (the authors of this study) administered the QLQ-C30 questionnaire by the European Organisation for Research and Treatment

	of Cancer (EORTC) and its breast cancer-specific module, QLQ-BR23. These validated instruments provided a reference (ground truth) against which the content of online discussions could be analyzed. The EORTC QLQ-C30 questionnaire was used to assess general QoL in cancer patients. It comprises 30 items grouped into five functioning scales, a global health status/QoL scale, three symptom scales, and six single-item measures. Scores range from 0 to 100, with higher functioning scores and lower symptom scores indicating better QoL. Normative values for this instrument have been previously established. The breast cancer-specific module, EORTC QLQ-BR23, consists of 23 items categorized into eight scales: systemic therapy side effects, hair loss, arm symptoms, breast symptoms, body image, future perspective, sexual functioning, and sexual enjoyment. Response options align with those of the EORTC QLQ-C30.<br>The final survey also included demographic questions such as year of birth, U.S. state of residence, gender, sex at birth, insurance type, and residential setting (urban, suburban, rural, or other).<br>Technical functionality has been tested by Inspire.com.
Open survey versus closed survey	Closed survey, all eligible community members with an account at Inspire.com were invited to participate (i.e., Convenience Sampling).
Contact mode	Initial contact was made via the Inspire.com website.
Advertising the survey	All eligible community members were invited to participate (i.e., Convenience Sampling), with the first 100 respondents receiving a $20 gift card.
Web/E-mail	The survey used a web site.
Context	The survey was conducted via Inspire.com, a patient community for patients of various diseases, e.g., breast cancer.
Mandatory/voluntary	Participation was voluntary.
Incentives	The first 100 respondents received a $20 gift card.
Time/Date	The survey was conducted between May and June 2024.
Randomization of items or questionnaires	Questionnaire items were presented in the standardized order of the EORTC QLQ-C30 and EORTC QLQ-BR23 questionnaires.
Adaptive questioning	Some questions in the questionnaire only had to be

	answered if a certain previous question was answered positively (e.g., a certain symptom is present).
Number of Items	The number of questionnaire items was 53 plus some questions about demographic data.
Number of screens (pages)	5
Completeness check	Completeness of mandatory items was checked using JavaScript.
Review step	There was no dedicated review step, but the main part of the survey was on a single page such that participants could review all their answers at all times.
Unique site visitor	A unique visitor was identified by its respective Inspire.com account.
View rate (Ratio of unique survey visitors/unique site visitors)	This data was not collected/analyzed.
Participation rate (Ratio of unique visitors who agreed to participate/unique first survey page visitors)	This data was not collected/analyzed.
Completion rate (Ratio of users who finished the survey/users who agreed to participate)	This data was not collected/analyzed.
Cookies used	Cookies were used for the standard Inspire.com login and used to identify unique users. These cookies were not saved for later analysis.
IP check	A unique participant was identified by its respective Inspire.com account.
Log file analysis	Log files were not analyzed for the purpose of this study.
Registration	The user registration was not specific to the survey but instead the regular Inspire.com accounts were used. Users could not participate multiple times. The username was not stored together with the survey results, but a generic user ID was saved, together with all posts of the respective user, following the purpose of the study to correlate these to the survey answers.
Handling of incomplete questionnaires	Incomplete questionnaires were not analyzed.
Questionnaires submitted with	No filtering based on time stamps/time frames was

an atypical timestamp	done.
Statistical correction	No such methods were applied.
